# Longitudinal Delta-Radiomics Identifies Treatment-Fraction-Associated Features Linked to Outcomes After Carbon-Ion Radiotherapy for Head and Neck Cancer

**DOI:** 10.3390/diagnostics16203309

**Published:** 2026-10-12

**Authors:** Xiangdi Meng, Zhuojun Ju, Nobuteru Kubo, Atsushi Musha, Tatsuya Ohno

**Affiliations:** 1 Department of Radiation Oncology, Gunma University Graduate School of Medicine, Maebashi 3718511, Japan; m2320043@gunma-u.ac.jp (X.M.);; 2 Department of Radiation Oncology, Weifang People’s Hospital, Weifang 261041, China; 3 Gunma University Heavy Ion Medical Center, Maebashi 3718511, Japan; 4 Department of Oral and Maxillofacial Surgery and Plastic Surgery, Gunma University Graduate School of Medicine, Maebashi 3718511, Japan

**Keywords:** carbon-ion radiotherapy, head and neck cancer, delta-radiomics, longitudinal imaging, prognosis

## Abstract

**Background**: Longitudinal imaging during carbon-ion radiotherapy (CIRT) may capture dose-related tumor evolution before clinical assessment. We investigated whether serial CT-based Δ-radiomic features follow treatment-fraction (dose escalation)-associated trajectories with prognostic relevance in patients with head and neck cancer (HNC) treated with CIRT. **Methods**: We analyzed 167 HNC patients receiving 16-fraction CIRT. Features were extracted from plan-CT, confirm-CT, and weekly-CT scans after 5, 9, and 13 fractions. Δ-radiomic features were defined as time-normalized relative changes from confirm-CT. Linear mixed-effects models, paired Wilcoxon signed-rank tests, and monotonicity assessments identified features with systematic longitudinal changes. Endpoints were progression-free survival (PFS), overall survival (OS), and local recurrence (LR). Optimal prediction timing was determined using optimism-corrected C-indices. Bootstrap resampling and multivariable Cox models assessed cutoff stability and incremental value beyond clinical factors. **Results**: During a median follow-up of 15.2 months (range: 1.2–67.7), 64 PFS events, 18 deaths, and 24 local recurrences occurred. Five, eight, and six candidate Δ-radiomic features were retained for PFS, OS, and LR. Selected prediction time points spanned fractions 5, 9, and 13. In multivariable analyses, four PFS-related features met the predefined robustness criteria and improved discrimination over the clinical model (C-index, 0.646–0.661 vs. 0.580; likelihood ratio test, *p* = 0.002–0.004). Sequential adjustment retained two OS-related and two LR-related features. Exploratory histology-specific analyses provided some support, primarily for two PFS-related features. **Conclusions**: Serial CT-based delta-radiomics identified treatment-fraction-associated imaging features associated with clinical endpoint risk after CIRT for HNC, particularly PFS. These findings are exploratory and require prospective external validation before clinical application.

## 1. Introduction

Head and neck cancer (HNC) remains challenging because of its biological heterogeneity, complex anatomy, and variable clinical course [1,2]. For selected patients with unresectable disease [3], radioresistant histologies, or tumors adjacent to critical normal structures [4,5,6], carbon-ion radiotherapy (CIRT) offers distinct physical and biological advantages [7,8]. However, predicting response to CIRT remains difficult, even among patients with the same tumor type or disease stage [8,9]. Previous prognostic studies of HNC treated with CIRT have primarily focused on clinical and histopathologic factors, whereas the prognostic relevance of longitudinal radiomic changes during CIRT remains poorly characterized. Prognostic assessment still relies largely on conventional clinical characteristics and long-term follow-up [4]. Candidate imaging features that identify patients likely to respond to CIRT could support early prognostic assessment and subsequent treatment and follow-up decisions.

Radiomics quantitatively characterizes tumor phenotypes from routinely acquired medical images and has shown promise for outcome prediction in HNC and other solid tumors [10,11,12,13]. Unlike baseline radiomics, delta-radiomics evaluates temporal changes in imaging features and may better represent treatment (dose)-related tumor evolution [14,15]. This longitudinal perspective is relevant to radiotherapy because tumor regression, structural remodeling, and other response patterns may develop before they become evident clinically [16,17]. Recent longitudinal radiomics studies in HNC have further demonstrated the potential value of serial imaging for outcome prediction [18]. However, most previous longitudinal delta-radiomic studies have involved photon-based treatment, used limited imaging time points and focused primarily on predictive performance rather than systematic trajectory characterization. Few studies have examined whether candidate features follow systematic treatment-related trajectories over time.

Serial CT acquisition during CIRT provides an opportunity to study temporal tumor evolution in HNC [19,20]. A clinically meaningful longitudinal imaging feature should demonstrate not only predictive association but also systematic treatment-associated evolution and outcome-specific trajectories [14,15]. This approach may distinguish biologically plausible response signals from stochastic variation and improve the interpretability of longitudinal radiomics. However, this concept remains insufficiently investigated in patients with HNC treated with CIRT. It remains unclear which Δ-radiomic features show reproducible treatment-fraction (dose escalation)-associated changes during CIRT, whether their trajectories associate with clinical outcomes, and when they are most prognostically informative.

Accordingly, we conducted a longitudinal delta-radiomic study using serial CT images acquired before and during CIRT for HNC. We aimed to identify treatment-fraction (dose escalation)-associated Δ-radiomic features with outcome-specific trajectories, determine their optimal prediction windows, and assess their incremental prognostic value beyond a prespecified clinical reference model. We hypothesized that longitudinal Δ-radiomic features may capture clinically relevant treatment-related changes and provide time-specific prognostic information beyond conventional clinical factors.

## 2. Materials and Methods

### 2.1. Patients

**Figure 1:**
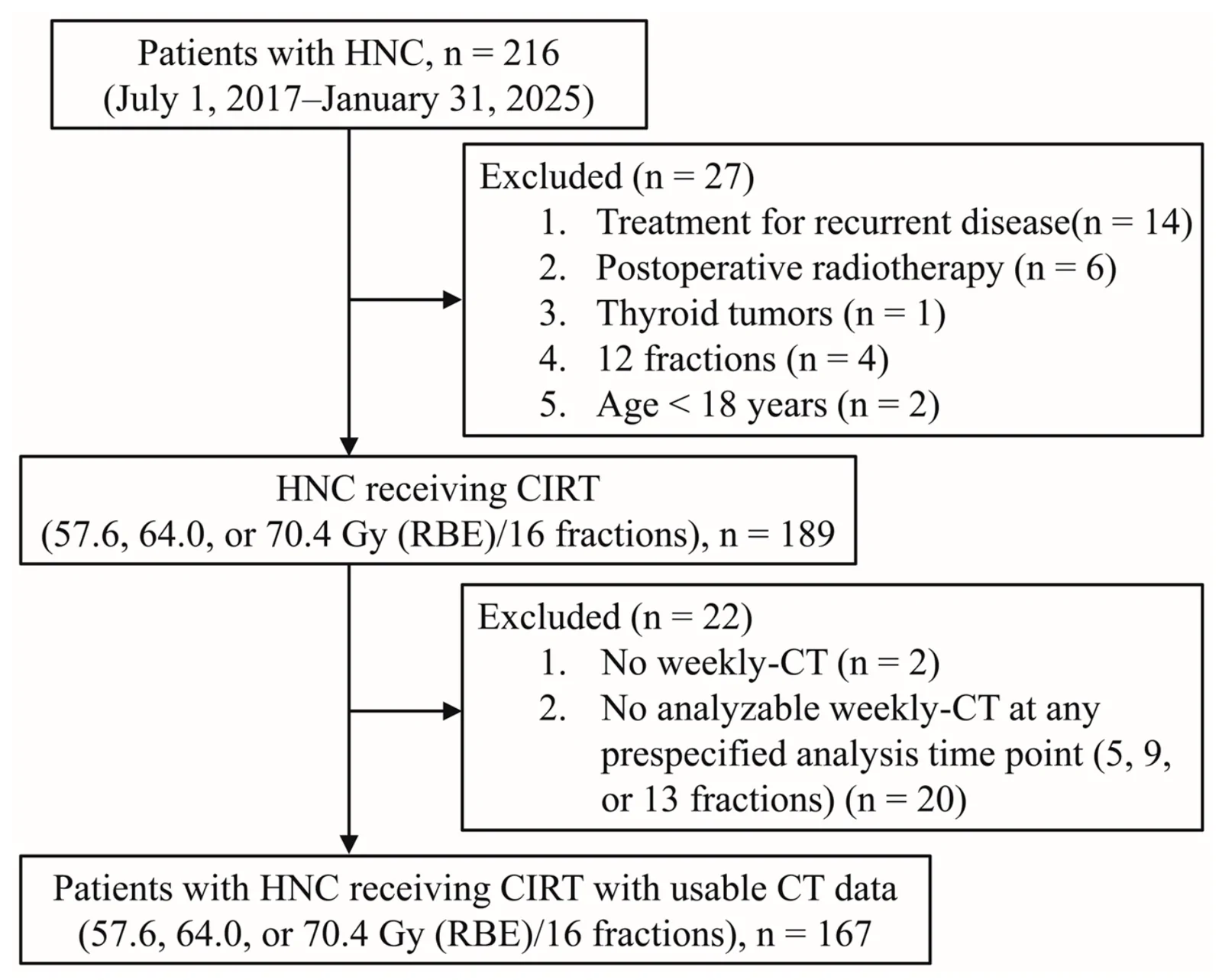
Patient-selection flowchart.

We retrospectively reviewed 216 consecutive patients with HNC who received CIRT at Gunma University Heavy Ion Medical Center between July 2017 and January 2025. We first excluded patients treated for recurrent disease or with postoperative radiotherapy, thyroid tumors, 12-fraction regimens, or age < 18 years. Among otherwise eligible patients, those without weekly-CT scans or without an analyzable weekly CT scan at any prespecified time point (after 5, 9, or 13 fractions) were subsequently excluded, leaving 167 patients for analysis. The patient-selection process is shown in Figure 1. Clinical records provided baseline data on sex, age, tumor site, histology, tumor stage, and prescribed radiation dose (Table 1). The CIRT procedure has been described previously [3,4,6,19,21,22]. All patients received CIRT in 16 fractions at prescribed doses of 57.6, 64.0, or 70.4 Gy (RBE), selected according to tumor type and proximity to organs at risk. The Gunma University institutional review board approved the study (No. HS2025-090; date: 14 July 2025) and waived the requirement for informed consent.

### 2.2. Image Acquisition

**Figure 2:**
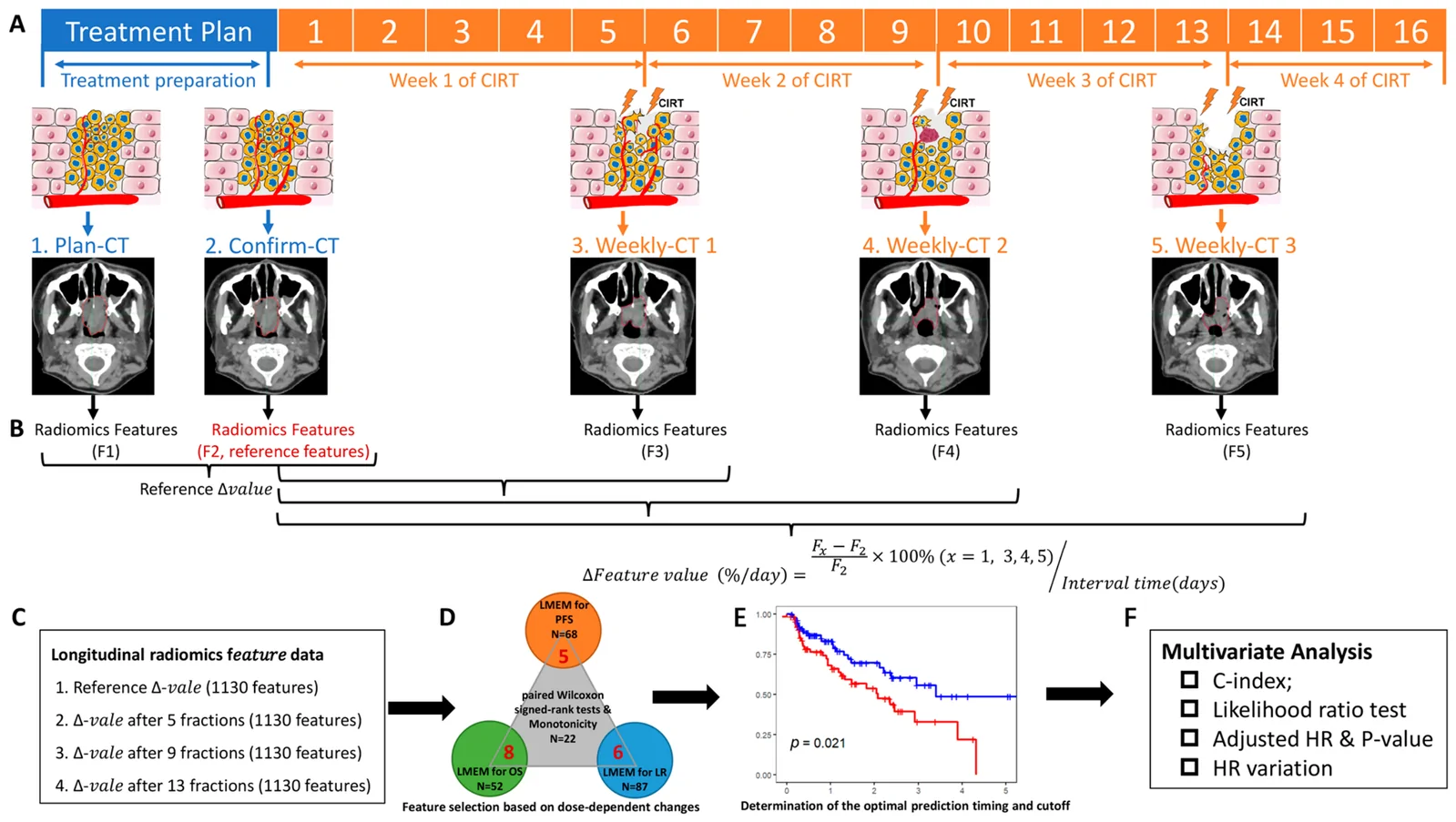
Study workflow. (**A**) Serial CT acquisition before and during CIRT; (**B**) computation of Δ-radiomic features; (**C**) longitudinal Δ-radiomic dataset; (**D**) identification of treatment-fraction-associated Δ-radiomic features using linear mixed-effects models, paired Wilcoxon signed-rank tests, and monotonicity assessment for PFS, OS, and LR; (**E**) determination of optimal prediction timing and cutoff values; and (**F**) multivariable Cox sensitivity analysis.

All patients underwent planning CT (plan-CT) scan 1–2 weeks before treatment. Confirmation CT (confirm-CT) was acquired 1–2 days before treatment to verify plan reproducibility and patient positioning. After treatment began, CT was performed weekly (weekly-CT; Figure 2A). Immobilization devices, patient positioning, and acquisition parameters were identical across plan-, confirm-, and weekly-CT scans. CT acquisition parameters are provided in Supplementary Table S1, and positioning methods have been described previously [3,4,5,6,19]. Tumor targets and organs at risk were delineated on plan-CT using MIM Maestro version 7.3.2 (MIM Software Inc., Cleveland, OH, USA). Confirm- and weekly-CT images were rigidly registered to plan-CT scans using bony anatomy, allowing propagation of the gross tumor volume (GTV) contour to each scan. All CT datasets were transferred to the XiO-N system (version 4.47; Elekta, Stockholm, Sweden; Mitsubishi Electric, Tokyo, Japan), where the original treatment plan was recalculated on confirm- and weekly-CT scans using identical planning parameters. Contours and dosimetric parameters were reviewed on confirm- and weekly-CT scans. When clinically relevant changes in tumor position, tumor volume, or dose constraints were identified, target contours and/or treatment plans were adapted as part of routine clinical care [19]. For Δ-feature analysis, we exported plan-CT (*n* = 167), confirm-CT (*n* = 167), and at least one weekly-CT scan per patient, including scans after 5 (*n* = 148), 9 (*n* = 147), and 13 (*n* = 72) fractions (Figure 2B). Weekly-CT scans acquired outside the prespecified 5-, 9-, or 13-fraction time points were excluded to maintain consistency in the fraction-based Δ-radiomic analysis. In addition, baseline clinical characteristics were compared between patients with and without analyzable weekly-CT scans at each prespecified time point to assess potential selection bias related to scan availability. The GTV served as the region of interest for radiomic feature extraction from each CT dataset.

### 2.3. Radiomic Feature Extraction and Delta-Feature Calculation

Radiomic features were extracted using in-house Python scripts (version 3.7.12) based on PyRadiomics version 3.1.0. Before extraction, CT images were resampled to isotropic 1 × 1 × 1 mm^3^ voxels using B-spline interpolation. For first-order and texture analyses, gray levels were discretized using a fixed bin width of 25 Hounsfield units. The feature set comprised 14 shape, 18 first-order, and 75 texture features: 24 gray-level co-occurrence matrix (GLCM), 16 gray-level run-length matrix (GLRLM), 16 gray-level size zone matrix (GLSZM), 14 gray-level dependence matrix (GLDM), and five neighboring gray-tone difference matrix (NGTDM) features. Shape features were extracted only from the original images, whereas first-order and texture features were extracted from 12 image types: original images, Laplacian-of-Gaussian-filtered images with sigma values of 1, 2, and 3 mm, and eight wavelet decompositions (LLL, LLH, LHL, LHH, HLL, HLH, HHL, and HHH) [13,23]. A total of 1130 radiomic features were generated per CT scan (Supplementary Table S2).

Using radiomic features from confirm-CT scans as the reference, Δ-features were defined as time-normalized relative changes (Figure 2B). Changes from plan- to confirm-CT scans represented pretreatment tumor evolution and served as reference Δ-features. Changes from confirm-CT to weekly-CT scans after 5, 9, and 13 fractions represented treatment-associated feature alterations during CIRT. Because scan intervals varied, each Δ-feature was normalized by the signed number of days relative to confirm-CT scans. Confirm-CT scans served as the temporal origin; pretreatment scans were assigned negative time values and on-treatment scans were assigned positive values. This transformation expressed Δ-features as time-normalized relative change rates on a common directional scale, avoiding artificial sign inversion between pretreatment and on-treatment changes. Four delta-radiomic feature datasets were generated (Figure 2C).

### 2.4. Statistical Analysis

#### 2.4.1. Delta-Radiomic Feature Selection

A two-step analysis identified radiomic features with systematic treatment-fraction-associated changes during CIRT and evaluated whether their trajectories differed by endpoint event status (Figure 2D). The strategy was designed to retain biologically plausible treatment-associated changes while limiting spurious fluctuations.

In step 1, a linear mixed-effects model (LMEM) with a patient-specific random intercept evaluated whether Δ-radiomic features changed systematically during treatment according to clinical outcome. This analysis identified treatment-related trajectories potentially associated with clinical outcomes rather than developing a prognostic model [16,24,25]. In contrast, conventional machine-learning and deep-learning approaches are typically based on fixed predictor sets and were therefore not considered in this study.

Δvalue ~ fraction×outcome+(1 | patient ID)

Here, Δvalue represents the time-normalized relative change rate of each feature relative to the confirm-CT baseline (Figure 2). Fraction denotes treatment stage at 0 (before treatment), 5, 9, or 13 fractions. Because all patients received 16-fraction CIRT, treatment fraction served as a clinically interpretable surrogate for cumulative exposure. Accordingly, the trajectories represent treatment-fraction-associated changes rather than absolute physical dose–response relationships. Outcome denotes event status for progression-free survival (PFS), overall survival (OS), or local recurrence (LR). PFS was defined as the interval from CIRT completion to disease progression, death, or last follow-up. OS was defined as the interval from CIRT completion to death from any cause or last follow-up. LR was defined as the interval from CIRT completion to local recurrence or last follow-up. Patients without the corresponding event were censored at the last follow-up. The term (1|*patient ID*) represents a patient-specific random intercept accounting for within-patient correlation from repeated measurements.

The LMEM yielded two *p* values: P_fraction and P_interaction. P_fraction tested the overall association between feature change and treatment stage. P_interaction tested whether trajectories differed by clinical outcome. Features with significant P_fraction and P_interaction were considered to show treatment-associated trajectories linked to clinical outcome. *p* values were adjusted for multiple comparisons using the Benjamini–Hochberg false discovery rate (BH-FDR) procedure, with adjusted *p* < 0.05 considered statistically significant [26].

In step 2, paired Wilcoxon signed-rank tests compared each pretreatment reference Δ-feature, defined as the time-normalized change from plan- to confirm-CT scans, with the corresponding on-treatment values after 5, 9, and 13 fractions. This analysis confirmed treatment-fraction-associated feature evolution after the trajectory analysis. Features with consistent significance across increasing fractions were considered treatment-associated. Features were excluded if significance at an earlier comparison was not maintained later or if no comparison was significant (0 vs. 5, 0 vs. 9, or 0 vs. 13 fractions). *p* values were adjusted using the BH-FDR procedure. Monotonicity was assessed by comparing median Δ-feature values at baseline and after 5, 9, and 13 fractions. Features with continuously increasing or decreasing trends were retained.

Features meeting both sets of criteria were retained as candidate treatment-fraction-associated Δ-radiomic features for survival analyses (Figure 2D).

#### 2.4.2. Optimal Prediction Timing for Each Feature

For each retained Δ-feature, univariable Cox proportional hazards models were fitted at 0, 5, 9, and 13 fractions for the corresponding endpoint (PFS, OS, or LR). The 0-fraction value represented the pretreatment reference Δ-feature derived from the change between plan- and confirm-CT scans. Model discrimination was quantified using Harrell’s concordance index (C-index). Internal validation used 1000 bootstrap resamples to calculate optimism-corrected C-indices at each time point. For each resample, optimism was estimated as the difference between performance in the bootstrap sample and in the original dataset, then subtracted from the apparent C-index [27,28].

$$C_{corrected\;}=C_{apparent}-\frac{1}{1000}\sum_{b=1}^{1000}\left(C_{bootstrap,b\;}-C_{original,b}\right)$$

where $C_{corrected\;}$ denotes the optimism-corrected C-index; $C_{apparent}$ denotes the apparent C-index; in the original dataset, $C_{bootstrap,b\;}$ denotes the C-index in bootstrap sample b; and $C_{original,b}$ denotes the C-index obtained by applying the model fitted in bootstrap sample b to the original dataset.

For each feature, the treatment stage with the highest optimism-corrected C-index was defined as the optimal prediction time. This approach prioritized model discrimination over statistical significance alone.

#### 2.4.3. Determination of Optimal Δ-Radiomic Feature Cutoffs

For each Δ-radiomic feature at its optimal prediction time, maximally selected log-rank statistics determined the cutoff separating high- and low-risk groups [29,30] (Figure 2E). This method identifies the threshold that maximizes survival-curve separation. Hazard ratios, *p* values, and optimism-corrected C-indices were estimated using the selected cutoff.

Cutoff stability was assessed using 1000 bootstrap iterations. The optimal cutoff was re-estimated in each bootstrap sample using the same procedure. Because cutoffs were re-estimated in each bootstrap sample, the resulting intervals were interpreted as stability measures rather than as direct counterparts to apparent *p* values. Cutoff-based stratification was considered robust when both apparent and bootstrap median Cox-model *p* values were <0.05 and the apparent HR fell within the bootstrap 2.5th–97.5th percentile interval.

#### 2.4.4. Multivariable Assessment of Incremental Prognostic Value

A prespecified clinical reference model (CM) included age, TNM stage (I–III vs. IV), and tumor histology as representative patient-, disease-, and tumor-related factors, respectively. The CM was designed as a clinically interpretable benchmark for assessing the incremental prognostic information of individual Δ-radiomic features. For PFS, each dichotomized Δ-radiomic feature was added individually to the CM at its optimal prediction time to form an extended model (EM).

Incremental prognostic value was assessed by comparing the CM and EM using C-indices and likelihood ratio tests (LRTs) (Figure 2F). Adjusted HR direction was compared with the univariable estimate to assess consistency. Adjustment-related variability was quantified as the relative change in log-HR (β) [31,32,33]:

Relative change in log(HR) = |β_adj − β_uni| / |β_uni| × 100%

where β = log(HR), β_uni is the univariable Cox coefficient, and β_adj is the multivariable coefficient. Internal validation used 1000 bootstrap resamples.

For OS and LR, sequentially adjusted models were used because of the smaller event counts. Given the fixed retrospective cohort, post hoc power was not calculated; uncertainty in these secondary analyses was instead assessed using effect estimates and bootstrap resampling [34]. Each Δ-feature was modeled separately with age, TNM stage, and histology, yielding three adjusted models. The same performance and robustness metrics were applied. Each Δ-feature model was compared with the corresponding single-variable clinical model using the C-index and LRT.

For PFS, a Δ-radiomic feature was supported if the adjusted and univariable HRs were directionally concordant [35], log-HR variation was <10% [31], both apparent and bootstrap median *p* values were <0.05 [36], and the EM improved the C-index and LRT relative to the CM. For OS and LR, all criteria had to be met across the three sequentially adjusted models.

#### 2.4.5. Histology-Specific Subgroup Sensitivity Analysis

Exploratory subgroup analyses assessed whether associations of retained Δ-radiomic features were consistent across the three most prevalent histologic subtypes: adenoid cystic carcinoma (ACC; *n* = 43), malignant melanoma (MM; *n* = 38), and squamous cell carcinoma (SCC; *n* = 20). The analyses examined whether the direction and overall pattern observed in the full cohort were preserved within each subgroup. For each retained endpoint-specific Δ-feature, the full-cohort cutoff was applied unchanged within each subgroup, followed by descriptive univariable Cox regression. Bootstrap resampling assessed estimate stability within each subgroup. Because subgroup sizes and event counts were limited, particularly for OS and LR, these analyses were descriptive and hypothesis-generating. Smaller histologic subtypes were not analyzed separately because of sparse events and limited power.

Radiomic features were extracted using Python 3.7.12 (Python Software Foundation, Beaverton, OR, USA), and statistical analyses were performed in R 4.4.0 (R Foundation for Statistical Computing, Vienna, Austria).

## 3. Results

### 3.1. Patient Characteristics

The study included 167 patients (Table 1). The median age was 70 years (interquartile range [IQR]: 56.5–77.0). At diagnosis, most patients had stage IV disease (67.1%; *n* = 112). The most common primary sites were the nasal cavity or paranasal sinuses (49.7%; *n* = 83) and salivary glands (24.0%; *n* = 40). The predominant histologic subtypes were adenoid cystic carcinoma (25.7%; *n* = 43), malignant melanoma (22.8%; *n* = 38), squamous cell carcinoma (12.0%; *n* = 20), mucoepidermoid carcinoma (7.2%; *n* = 12), and sarcoma (4.8%; *n* = 8). All patients completed 16-fraction CIRT. Follow-up began after CIRT completion. The median follow-up was 15.2 months (range: 1.2–67.7). During follow-up, 64 PFS events occurred. Eighteen patients (10.8%) died; local recurrence, lymph node metastasis, and distant metastasis occurred in 24 (14.4%), 22 (13.2%), and 36 (21.6%) patients, respectively. These events were not mutually exclusive. No statistically significant differences in baseline clinical characteristics were observed between patients with and without analyzable weekly-CT scans at fractions 5, 9, or 13 (all *p* > 0.05; Supplementary Table S3).

### 3.2. Feature Selection

In the LMEM analysis, 68, 52, and 87 Δ-radiomic features showed significant associations with treatment fraction and significant fraction-by-event-status interactions for PFS, OS, and LR, respectively (BH-adjusted P_fraction and P_interaction < 0.05; Supplementary Table S4). These features therefore showed treatment-fraction-associated trajectories that differed by clinical outcome.

Paired Wilcoxon signed-rank tests compared Δ-feature values after 5, 9, or 13 fractions with pretreatment reference values. Overall, 175 Δ-radiomic features showed continuous or partially continuous differences from baseline (BH-adjusted *p* < 0.05). Monotonicity was assessed using median Δ-feature values across treatment stages. Forty-three Δ-radiomic features showed consistent monotonic trajectories across fractions. Twenty-two Δ-radiomic features met both the Wilcoxon and monotonicity criteria (Supplementary Table S5).

Integrating all three selection steps retained five, eight, and six candidate Δ-radiomic features for PFS, OS, and LR, respectively. These features showed systematic, monotonic treatment-fraction-associated changes and outcome-specific trajectories. Spearman correlation analysis showed that four of the five PFS-related features were moderately to strongly correlated (ρ = 0.78–1.00; Supplementary Figure S1). Among the eight OS-related features, three were highly correlated (ρ ≈ 1), whereas the others showed weaker correlations. Because each Δ-radiomic feature was evaluated separately in multivariable models, none was excluded solely because of inter-feature correlation.

### 3.3. Optimal Prediction Time Windows and Cutoff Determination

At each optimal prediction time, Δ-feature values were dichotomized using fraction-specific cutoffs. Most cutoff-based stratifications showed outcome discrimination, with generally consistent results across bootstrap resamples in univariable Cox analyses (Table 2). Two LR-related features did not meet all predefined robustness criteria. For Δ-wavelet.HHL_glcm_Idmn, the apparent *p* value was 0.032, but the bootstrap median *p* value was 0.073. For Δ-log.sigma.2.mm.3D_gldm_LargeDependenceHighGrayLevelEmphasis, the apparent *p* value was 0.066, and the apparent HR of 2.30 fell outside the bootstrap 2.5th–97.5th percentile interval of 2.68–5.37.

### 3.4. Multivariable Analysis

For PFS, each dichotomized Δ-radiomic feature was added individually to the prespecified clinical model of age, TNM stage, and histology at its optimal prediction time (fraction). All five candidate features increased the C-index relative to the clinical reference model and yielded significant LRT results after adjustment (Table 3). Four features met the predefined robustness criteria. The remaining feature, log.sigma.1.mm.3D_ngtdm_Strength, was unsupported because its bootstrap median log-HR variation exceeded the 10% threshold (14.91%).

Two Δ-radiomic features met the predefined robustness criteria for each of OS and LR. For OS, Δ-wavelet.HLL_glrlm_LongRunLowGrayLevelEmphasis and Δ-original_ngtdm_Strength showed concordant HR directions, a log-HR variation of <10%, significant apparent and bootstrap median *p* values, and improved model performance across sequential adjustments. For LR, Δ-wavelet.LLL_glszm_LowGrayLevelZoneEmphasis and Δ-wavelet.LLL_ngtdm_Strength met the same criteria. The remaining features were unsupported because of inconsistent apparent and bootstrap evidence, unstable HRs, or insufficient improvement across adjusted models.

Supported Δ-radiomic features were predominantly texture-based. PFS-related features included GLRLM-, GLDM-, and GLCM-based measures, whereas OS- and LR-related features included GLRLM-, GLSZM-, and NGTDM-based measures. This pattern suggests that longitudinal texture changes contributed across different clinical endpoints, although the specific retained features differed by outcome.

### 3.5. Histology-Specific Subgroup Sensitivity Analysis

In exploratory histology-specific subgroup analyses (Supplementary Figure S2), two supported PFS-related Δ-radiomic features—log.sigma.1.mm.3D_glrlm_LongRunHighGrayLevelEmphasis and log.sigma.1.mm.3D_gldm_LargeDependenceHighGrayLevelEmphasis—showed directionally consistent, nominally significant associations with PFS across ACC, MM, and SCC (Supplementary Figure S2). These subgroup findings require cautious interpretation because event counts were small, particularly in MM and SCC. The other supported PFS-related features yielded unstable or off-scale estimates, particularly in MM and SCC, precluding reliable subgroup inference.

For OS, subgroup support was limited due to the small sample size: only Δ-wavelet.HLL_glrlm_LongRunLowGrayLevelEmphasis showed a nominally significant association in ACC, whereas the remaining estimates were unstable or non-estimable because of sparse events. For LR, only Δ-wavelet.LLL_glszm_LowGrayLevelZoneEmphasis showed a nominally significant association in ACC; estimates for the other retained feature were unstable or non-estimable. Overall, the subgroup analyses provided only partial, descriptive support, with the greatest consistency observed for the two PFS-related features.

## 4. Discussion

In this longitudinal CT-based delta-radiomic study of patients with HNC treated with CIRT, we identified imaging features with systematic treatment-fraction (dose escalation)-associated changes and outcome-specific trajectories. Their prognostic relevance varied across treatment time points, with optimal prediction times after 5, 9, and 13 fractions, and the strongest and most consistent incremental prognostic information was observed for PFS. Unlike most previous HNC radiomic studies that have focused on pretreatment imaging or isolated paired comparisons [13,15,37,38], our framework required longitudinal regularity, treatment-fraction association, and outcome-related trajectory differences before survival modeling, thereby reducing the likelihood that retained features reflected random variation, contouring effects, or nonspecific treatment-related fluctuation. This approach also extends longitudinal delta-radiomic research in CIRT, which remains less well characterized than in photon-based radiotherapy [18].

The retained features were predominantly texture-based, suggesting that longitudinal textural variation during CIRT may be clinically relevant. Because Δ-features were defined as time-normalized relative changes from confirm-CT scans, they reflected the direction and magnitude of treatment-associated feature changes. The retained PFS-related features, including LoG-derived GLRLM_LongRunHighGrayLevelEmphasis and GLDM_LargeDependenceHighGrayLevelEmphasis, increased during CIRT. HRs of >1 indicated that higher Δ-values were associated with greater progression risk [39,40,41]. In contrast, the retained OS- and LR-related features, including wavelet-derived GLRLM_LongRunLowGrayLevelEmphasis and GLSZM_LowGrayLevelZoneEmphasis, decreased during CIRT and had negative cutoffs. HRs of >1 indicated that higher and less negative Δ-values were associated with poorer survival and greater local recurrence risk, respectively [42,43,44]. Together, these findings suggest that multiscale longitudinal texture changes may carry prognostic information. The retained features quantify changes in the spatial organization of gray levels rather than specific biological processes; therefore, their longitudinal trajectories may reflect changes in intratumoral imaging heterogeneity [15], but the underlying biological basis cannot be determined from CT radiomics alone [16]. The different trajectory directions observed for PFS, OS, and LR are not necessarily contradictory because different texture features were retained for different clinical endpoints. Given the limited sample size, these findings remain exploratory and require validation in larger independent cohorts.

The time-specific findings may also have clinical relevance. The distribution of optimal prediction windows across 5, 9, and 13 fractions suggests that on-treatment imaging time points differ in prognostic value, consistent with previous delta-radiomics studies [16,45]. If externally validated, these time-specific imaging features may inform on-treatment risk stratification and help clinicians identify patients with unfavorable imaging trajectories who may benefit from earlier reassessment and closer post-CIRT surveillance. Although the extended models showed improved within-cohort discrimination over the clinical reference model, the magnitude of improvement was modest, and their clinical utility remains to be established through prospective external validation.

Several methodological features strengthen the interpretability of the findings. Serial CT acquired before and during CIRT enabled longitudinal assessment of treatment-related imaging changes, while confirm-CT provided a common temporal reference and captured early pre-irradiation variation [20]. Feature selection required treatment-fraction-associated change, longitudinal regularity, monotonicity, and outcome association, thereby reducing spurious findings in high-dimensional analyses [16]. Bootstrap-based internal assessment of prediction timing, cutoff stability, and multivariable performance provided additional evidence of internal consistency [26,27,28]. However, the bootstrap procedures did not repeat the entire upstream feature-selection process within each resample; therefore, residual selection-related optimism cannot be excluded, and nested or fully resampled validation should be considered in future studies.

Several limitations of the study should be considered. First, the retrospective single-center design may limit generalizability. Second, heterogeneity in primary site and histology reflected real-world CIRT practice but may have influenced radiomic behavior. Consistent with this concern, after subgroup analyses according to histology, the number of features that remained significant in Cox regression decreased from eight to four, suggesting that part of the radiomic–outcome association may be influenced by histological heterogeneity. Although SCC predominates in the general HNC population, non-squamous histologies are common among patients selected for CIRT; SCC accounted for only 6.9% in the multicenter J-CROS 1402 HN cohort (*n* = 908) and 5.1% in a previous CIRT series of 236 patients [8,46]. The patient distribution in our cohort was consistent with previous CIRT studies, whereas the applicability of our findings to photon- or proton-treated populations requires further validation. In addition, dose-stratified analyses were not performed because prescribed dose was histology- and protocol-dependent and markedly imbalanced across groups (57.6 Gy [RBE], *n* = 34; 64.0 Gy [RBE], *n* = 124; 70.4 Gy [RBE], *n* = 9), precluding reliable separation of dose-related effects from histologic differences. Third, the limited numbers of OS and LR events, together with the relatively short median follow-up of 15.2 months, constrain the maturity of these secondary endpoint analyses, particularly OS, and warrant cautious interpretation. Fourth, only weekly CT scans acquired at the prespecified 5-, 9-, and 13-fraction time points were included to ensure temporal consistency. Because some patients were scanned at other fractions, such as 5, 9, and 14, their nonmatching scans were excluded from time-specific analyses. This reduced the number of analyzable datasets from fraction 13 to 72 and may have affected estimate stability, particularly for OS and LR, given the limited numbers of events at this time point. Although no statistically significant differences in baseline characteristics were observed between patients with and without analyzable CT scans (Supplementary Table S3), residual selection bias cannot be excluded. Fifth, no shape features met the predefined selection and robustness criteria. Segmentation-related variability cannot be excluded because, except during adaptive replanning, the GTV was propagated to weekly CT scans by rigid registration rather than manually recontoured [19]. This approach may have introduced variability in longitudinal shape measurements. Shape features may also change non-monotonically during radiotherapy, for example through transient enlargement followed by shrinkage, making them less suitable for the treatment-fraction-associated framework used here. Additionally, because adaptive contour or plan adjustments were part of routine clinical management, their potential influence on Δ-radiomic trajectories was not evaluated separately in the present study. Sixth, radiomic features may be influenced by image acquisition, registration, contour propagation, preprocessing, and feature-definition choices. Test–retest or interobserver repeatability filtering was not performed, and future external validation should incorporate repeatability-based feature prefiltering. Although the Δ-features retained directional information, their biological interpretation remains uncertain. Finally, bootstrap resampling assessed internal stability but did not repeat upstream feature selection or prediction-time selection; cutoffs were re-estimated at the selected time points. Therefore, it did not fully correct for selection-related optimism arising from feature selection, prediction-time selection, and cutoff determination. The reported performance estimates and cutoffs should be interpreted as exploratory within-cohort estimates rather than externally validated predictive estimates. Accordingly, the identified optimal time windows and cutoff values should not be extrapolated to other centers without prospective external validation.

## 5. Conclusions

Serial CT-based delta-radiomics identified treatment-fraction-associated Δ-radiomic features with the most consistent incremental prognostic associations for PFS, whereas the findings for OS and LR remain exploratory and hypothesis-generating because of the limited number of events. External validation in larger independent cohorts is required before clinical use.

## Figures and Tables

**Table 1:** Baseline characteristics of the study cohort (*n* = 167).

Characteristic	Overall Cohort, *n* (%)
Age, years, median [IQR]	70.0 [56.5–77.0]
Sex	
Female	71 (42.5)
Male	96 (57.5)
TNM stage	
I	4 (2.4)
II	12 (7.2)
III	39 (23.4)
IV	112 (67.1)
Primary tumor site	
Nasal cavity/paranasal sinuses	83 (49.7)
Salivary glands	40 (24.0)
Ear	9 (5.4)
Oral cavity	10 (6.0)
Upper/lower jaw	7 (4.2)
Orbit/skull base	2 (1.2)
Pharynx	9 (5.4)
Other sites	7 (4.2)
Histologic subtype	
Adenoid cystic carcinoma	43 (25.7)
Malignant melanoma	38 (22.8)
Squamous cell carcinoma	20 (12.0)
Mucoepidermoid carcinoma	12 (7.2)
Sarcoma	8 (4.8)
Other histologic subtypes	46 (27.5)
Total prescribed dose, Gy (RBE), delivered in 16 fractions	
57.6	34 (20.4)
64.0	124 (74.3)
70.4	9 (5.4)

Abbreviations: IQR, interquartile range; RBE, relative biological effectiveness.

**Table 2:** Threshold-based stratification of Δ-radiomic features using maximally selected log-rank statistics and univariable Cox regression.

Outcome	Δ-Radiomic Feature	Optimal Prediction Time, Fraction	Cutoff Value (%/Day), Apparent (Bootstrap Median)	Apparent HR (Bootstrap 2.5th–97.5th Percentile Interval)	Apparent *p* Value (Bootstrap Median)	Optimism-Corrected C-Index (Estimated Optimism)
PFS	log.sigma.1.mm.3D_glrlm_LongRunHighGrayLevelEmphasis	9	1.50 (1.37)	2.74 (2.61–4.33)	0.001 (0.001)	0.644 (0.023)
	log.sigma.1.mm.3D_gldm_LargeDependenceHighGrayLevelEmphasis	9	1.43 (1.34)	2.51 (2.42–4.13)	0.002 (0.001)	0.645 (0.021)
	log.sigma.1.mm.3D_glcm_Autocorrelation	9	−2.46 (−1.37)	5.89 (3.07–7.75)	0.014 (0.008)	0.629 (0.018)
	log.sigma.1.mm.3D_glcm_JointAverage	9	−1.13 (−0.63)	5.89 (2.96–7.43)	0.014 (0.008)	0.629 (0.018)
	log.sigma.1.mm.3D_ngtdm_Strength	5	−0.17 (−0.17)	1.80 (1.79–2.59)	0.038 (0.016)	0.639 (0.029)
OS	log.sigma.1.mm.3D_glcm_Autocorrelation	13	−1.25 (−0.86)	0.16 (0.07–0.26)	0.009 (0.040)	0.568 (0.032)
	wavelet.HLL_glrlm_LongRunLowGrayLevelEmphasis	13	−0.10 (−0.10)	4.30 (2.92–8.24)	0.018 (0.019)	0.629 (0.024)
	original_ngtdm_Strength	5	0.43 (0.43)	4.47 (3.35–8.41)	0.010 (0.012)	0.679 (0.030)
	original_shape_Maximum2DDiameterColumn	5	−1.03 (−0.83)	0.08 (0.06–0.20)	<0.001 (<0.001)	0.673 (0.021)
	wavelet.LHH_glcm_Correlation	9	0.94 (1.07)	3.85 (3.46–8.15)	0.015 (0.005)	0.641 (0.029)
	log.sigma.1.mm.3D_glcm_SumAverage	13	−0.46 (−0.41)	0.22 (0.08–0.30)	0.015 (0.033)	0.563 (0.022)
	log.sigma.1.mm.3D_glcm_JointAverage	13	−0.46 (−0.41)	0.22 (0.08–0.29)	0.015 (0.040)	0.561 (0.024)
	wavelet.HLL_gldm_LowGrayLevelEmphasis	9	1.80 (0.70)	3.54 (2.94–7.22)	0.044 (0.036)	0.589 (0.029)
LR	wavelet.LLL_glszm_LowGrayLevelZoneEmphasis	13	−1.38 (−1.38)	3.82 (3.49–6.03)	0.041 (0.038)	0.641 (0.014)
	wavelet.LLL_ngtdm_Strength	9	−1.31 (−0.22)	3.99 (2.66–4.01)	0.043 (0.041)	0.604 (0.012)
	wavelet.LLH_glcm_MCC	5	−1.43 (−0.45)	0.27 (0.23–0.37)	0.007 (0.018)	0.609 (0.027)
	wavelet.HHL_glcm_Idmn	13	0.01 (0.00)	3.64 (2.56–7.54)	0.032 (0.073)	0.572 (0.019)
	wavelet.LHH_gldm_DependenceNonUniformityNormalized	13	0.44 (0.43)	3.56 (1.65–5.79)	0.020 (0.015)	0.607 (0.027)
	log.sigma.2.mm.3D_gldm_LargeDependenceHighGrayLevelEmphasis	9	1.17 (0.09)	2.30 (2.68–5.37)	0.066 (0.031)	0.610 (0.025)

Values outside parentheses are apparent estimates from the original dataset. Values in parentheses are bootstrap-derived estimates from 1000 resamples: medians for cutoff values and *p* values, 2.5th–97.5th percentile intervals for HRs, and estimated optimism for C-indices. Because apparent estimates and bootstrap-derived intervals were obtained using different frameworks, the apparent HR and *p* value may not correspond exactly to the bootstrap percentile interval. Abbreviations: HR, hazard ratio; LR, local recurrence; OS, overall survival; PFS, progression-free survival.

**Table 3:** Multivariable assessment of the incremental prognostic value of Δ-radiomic features.

Outcome	Δ-Radiomic Feature	Optimal Prediction Time, Fraction	Clinical Model	CM Optimism-Corrected C-Index (Estimated Optimism)	EM Optimism-Corrected C-Index (Estimated Optimism)	LRT Apparent *p* Value (Bootstrap Median)	Feature Apparent HR (Bootstrap 2.5th–97.5th Percentile Interval)	Feature Apparent Log-HR Variation, % (Bootstrap Median)	Feature Apparent *p* Value (Bootstrap Median)
PFS	**log.sigma.1.mm.3D_glrlm_LongRunHighGrayLevelEmphasis**	9	Age + TNM stage + histology	0.580 (0.028)	0.650 (0.018)	0.003 (0.001)	2.74 (2.69–4.94)	0.14 (4.50)	0.002 (0.002)
	**log.sigma.1.mm.3D_gldm_LargeDependenceHighGrayLevelEmphasis**	9		0.580 (0.028)	0.661 (0.019)	0.004 (0.001)	2.55 (2.21–4.72)	1.54 (3.21)	0.003 (0.002)
	**log.sigma.1.mm.3D_glcm_Autocorrelation**	9		0.580 (0.028)	0.648 (0.038)	0.002 (0.001)	6.14 (3.24–9.91)	2.38 (2.56)	0.017 (0.008)
	**log.sigma.1.mm.3D_glcm_JointAverage**	9		0.580 (0.028)	0.646 (0.025)	0.002 (0.001)	6.14 (3.06–8.79)	2.38 (9.74)	0.017 (0.009)
	log.sigma.1.mm.3D_ngtdm_Strength	5		0.56 (0.015)	0.664 (0.019)	0.026 (0.011)	1.89 (1.85–2.91)	8.27 (14.91)	0.029 (0.013)
OS	log.sigma.1.mm.3D_glcm_Autocorrelation	13	Age	0.521 (0.029)	0.614 (0.024)	0.028 (0.018)	0.17 (0.07–0.29)	2.87 (3.25)	0.011 (0.053)
			TNM stage	0.576 (0.032)	0.618 (0.028)	0.020 (0.013)	0.15 (0.02–0.27)	4.59 (8.87)	0.007 (0.055)
			Histology	0.520 (0.014)	0.621 (0.018)	0.036 (0.035)	0.15 (0.03–0.29)	3.87 (12.98)	0.020 (0.096)
	**wavelet.HLL_glrlm_LongRunLowGrayLevelEmphasis**	13	Age	0.521 (0.029)	0.572 (0.023)	0.022 (0.015)	4.49 (3.40–8.59)	3.96 (4.09)	0.028 (0.029)
			TNM stage	0.576 (0.032)	0.633 (0.017)	0.023 (0.015)	4.07 (2.72–9.16)	3.82 (5.76)	0.030 (0.040)
			Histology	0.520 (0.014)	0.590 (0.018)	0.038 (0.024)	3.91 (2.41–10.89)	6.51 (7.28)	0.040 (0.047)
	**original_ngtdm_Strength**	5	Age	0.594 (0.008)	0.710 (0.009)	0.005 (0.003)	4.49 (3.40–8.59)	0.16 (2.71)	0.010 (0.011)
			TNM stage	0.544 (0.030)	0.692 (0.005)	0.004 (0.002)	4.59 (3.50–8.93)	1.69 (4.33)	0.008 (0.009)
			Histology	0.514 (0.010)	0.643 (0.012)	0.003 (0.002)	5.13 (3.63–12.42)	9.07 (9.42)	0.007 (0.010)
	original_shape_Maximum2DDiameterColumn	5	Age	0.594 (0.008)	0.730 (0.026)	<0.001 (0.001)	0.09 (0.06–0.22)	2.73 (3.82)	<0.001 (<0.001)
			TNM stage	0.544 (0.030)	0.700 (0.024)	<0.001 (0.002)	0.09 (0.06–0.24)	5.80 (7.63)	<0.001 (0.001)
			Histology	0.514 (0.010)	0.609 (0.017)	<0.001 (0.001)	0.06 (0.04–0.16)	11.11 (12.40)	<0.001 (<0.001)
	wavelet.LHH_glcm_Correlation	9	Age	0.534 (0.031)	0.638 (0.021)	0.013 (0.004)	3.76 (3.31–8.43)	1.68 (2.98)	0.017 (0.007)
			TNM stage	0.525 (0.016)	0.616 (0.022)	0.009 (0.004)	4.25 (3.69–8.28)	7.45 (5.94)	0.012 (0.005)
			Histology	0.580 (0.023)	0.645 (0.021)	0.033 (0.010)	3.22 (2.73–8.46)	13.30 (13.23)	0.039 (0.015)
	log.sigma.1.mm.3D_glcm_SumAverage	13	Age	0.521 (0.039)	0.541 (0.013)	0.030 (0.018)	0.22 (0.07–0.31)	1.70 (3.60)	0.017 (0.045)
			TNM stage	0.576 (0.032)	0.635 (0.019)	0.027 (0.017)	0.21 (0.05–0.32)	0.47 (9.29)	0.015 (0.049)
			Histology	0.520 (0.014)	0.522 (0.019)	0.026 (0.030)	0.17 (0.04–0.32)	13.79 (19.52)	0.020 (0.078)
	log.sigma.1.mm.3D_glcm_JointAverage	13	Age	0.521 (0.029)	0.532 (0.012)	0.030 (0.019)	0.22 (0.07–0.32)	1.70 (3.97)	0.017 (0.049)
			TNM stage	0.576 (0.032)	0.634 (0.020)	0.027 (0.018)	0.21 (0.05–0.32)	0.47 (9.20)	0.015 (0.058)
			Histology	0.520 (0.014)	0.517 (0.024)	0.026 (0.036)	0.17 (0.04–0.33)	13.79 (19.92)	0.020 (0.108)
	wavelet.HLL_gldm_LowGrayLevelEmphasis	9	Age	0.534 (0.031)	0.546 (0.021)	0.048 (0.024)	3.97 (2.98–8.32)	9.02 (4.22)	0.032 (0.036)
			TNM stage	0.525 (0.016)	0.515 (0.017)	0.054 (0.029)	3.75 (2.72–7.63)	4.52 (5.03)	0.036 (0.048)
			Histology	0.580 (0.023)	0.532 (0.022)	0.190 (0.042)	2.59 (2.53–8.52)	27.81 (18.60)	0.172 (0.085)
LR	**wavelet.LLL_glszm_LowGrayLevelZoneEmphasis**	13	Age	0.502 (0.018)	0.562 (0.019)	0.025 (0.013)	3.79 (3.37–6.27)	0.63 (2.18)	0.043 (0.047)
			TNM stage	0.654 (0.012)	0.703 (0.012)	0.011 (0.010)	4.83 (3.47–7.81)	1.49 (1.24)	0.024 (0.036)
			Histology	0.646 (0.014)	0.666 (0.018)	0.026 (0.010)	4.11 (2.90–7.60)	1.43 (1.72)	0.038 (0.031)
	**wavelet.LLL_ngtdm_Strength**	9	Age	0.501 (0.025)	0.569 (0.020)	0.023 (0.011)	4.09 (2.70–6.10)	1.83 (1.30)	0.038 (0.049)
			TNM stage	0.544 (0.026)	0.572 (0.025)	0.026 (0.011)	4.04 (2.74–6.12)	0.83 (5.28)	0.033 (0.041)
			Histology	0.607 (0.021)	0.626 (0.011)	0.018 (0.009)	4.39 (3.13–7.55)	6.92 (7.62)	0.049 (0.040)
	wavelet.LLH_glcm_MCC	5	Age	0.504 (0.019)	0.606 (0.017)	0.014 (0.019)	0.26 (0.22–2.63)	1.65 (2.73)	0.060 (0.018)
			TNM stage	0.552 (0.021)	0.608 (0.026)	0.022 (0.024)	0.28 (0.20–2.16)	5.00 (14.41)	0.011 (0.118)
			Histology	0.591 (0.023)	0.620 (0.025)	0.006 (0.010)	0.20 (0.17–3.27)	20.48 (23.41)	0.002 (0.068)
	wavelet.HHL_glcm_Idmn	13	Age	0.502 (0.018)	0.544 (0.016)	0.040 (0.032)	4.07 (2.81–9.98)	8.70 (7.90)	0.023 (0.062)
			TNM stage	0.654 (0.012)	0.658 (0.011)	0.143 (0.076)	2.61 (2.04–7.62)	25.59 (18.14)	0.118 (0.108)
			Histology	0.646 (0.014)	0.699 (0.023)	0.021 (0.015)	5.95 (3.63–22.94)	38.07 (33.62)	0.012 (0.036)
	wavelet.LHH_gldm_DependenceNonUniformityNormalized	13	Age	0.502 (0.018)	0.536 (0.022)	0.030 (0.022)	3.67 (0.66–6.68)	2.27 (6.77)	0.024 (0.018)
			TNM stage	0.654 (0.012)	0.693 (0.018)	0.011 (0.007)	4.49 (0.68–7.81)	18.13 (21.44)	0.007 (0.006)
			Histology	0.646 (0.014)	0.679 (0.020)	0.010 (0.010)	6.22 (0.74–10.84)	43.84 (36.45)	0.007 (0.009)
	log.sigma.2.mm.3D_gldm_LargeDependenceHighGrayLevelEmphasis	9	Age	0.501 (0.025)	0.538 (0.018)	0.080 (0.015)	2.68 (2.65–5.35)	0.74 (1.71)	0.068 (0.032)
			TNM stage	0.544 (0.026)	0.581 (0.018)	0.062 (0.015)	2.74 (2.62–5.76)	7.25 (7.28)	0.051 (0.039)
			Histology	0.607 (0.021)	0.657 (0.026)	0.013 (0.005)	3.76 (3.38–9.04)	59.16 (23.83)	0.010 (0.013)

Features meeting the predefined robustness criteria are shown in bold. Values outside parentheses are apparent estimates from the original dataset. Values in parentheses are bootstrap-derived estimates from 1000 resamples: medians for *p* values, HR variation, and optimism, and 2.5th–97.5th percentile intervals for HRs. Because apparent estimates and bootstrap-derived intervals were obtained using different frameworks, the apparent HR and *p* value may not correspond exactly to the bootstrap percentile interval. Abbreviations: CM, clinical model; EM, extended model; HR, hazard ratio; LR, local recurrence; LRT, likelihood ratio test; OS, overall survival; PFS, progression-free survival.

## Data Availability

The raw data supporting the conclusions of this article will be made available by the authors on request.

## Supplementary Materials

The following supporting information can be downloaded at: https://www.mdpi.com/article/10.3390/diagnostics16203309/s1, Table S1: CT image acquisition parameters; Table S2: Radiomics features used in this study (*n* = 1130); Table S3: Comparison of baseline characteristics according to weekly-CT availability at prespecified treatment fractions; Table S4: Linear mixed-effects model results evaluating treatment-stage-associated Δ-radiomic feature trajectories and endpoint-specific event-status interactions; Table S5: Paired Wilcoxon signed-rank test and monotonicity assessment for treatment-stage-associated Δ-radiomic feature evolution; Figure S1: Correlation heatmap of treatment-fraction-related Δ-radiomic features; Figure S2: Exploratory histology-specific subgroup sensitivity analysis of supported Δ-radiomic features.

## Abbreviations

The following abbreviations are used in this manuscript:

CIRT	carbon-ion radiotherapy
HNC	head and neck cancer
PFS	progression-free survival
OS	overall survival
LR	local recurrence
RBE	relative biological effectiveness
GTV	gross tumor volume
GLCM	gray-level co-occurrence matrix
GLRLM	gray-level run-length matrix
GLSZM	gray-level size zone matrix
GLDM	gray-level dependence matrix
NGTDM	neighboring gray-tone difference matrix
LMEM	linear mixed-effects model
CM	clinical reference model
EM	extended model
LRT	likelihood ratio test
ACC	adenoid cystic carcinoma
MM	malignant melanoma
SCC	squamous cell carcinoma
BH-FDR	Benjamini–Hochberg false discovery rate
