# Supplementary material for: Longitudinal Delta-Radiomics Identifies Treatment-Fraction-Associated Features Linked to Outcomes After Carbon-Ion Radiotherapy for Head and Neck Cancer
Source: Diagnostics (Basel). 2026 Oct 12;16(20):3309. doi: 10.3390/diagnostics16203309 (PMC13703572; doi:10.3390/diagnostics16203309)
Supplement: Supplementary file 1 [file diagnostics-16-03309-s001.zip › Supplementary Figure S1.pdf]

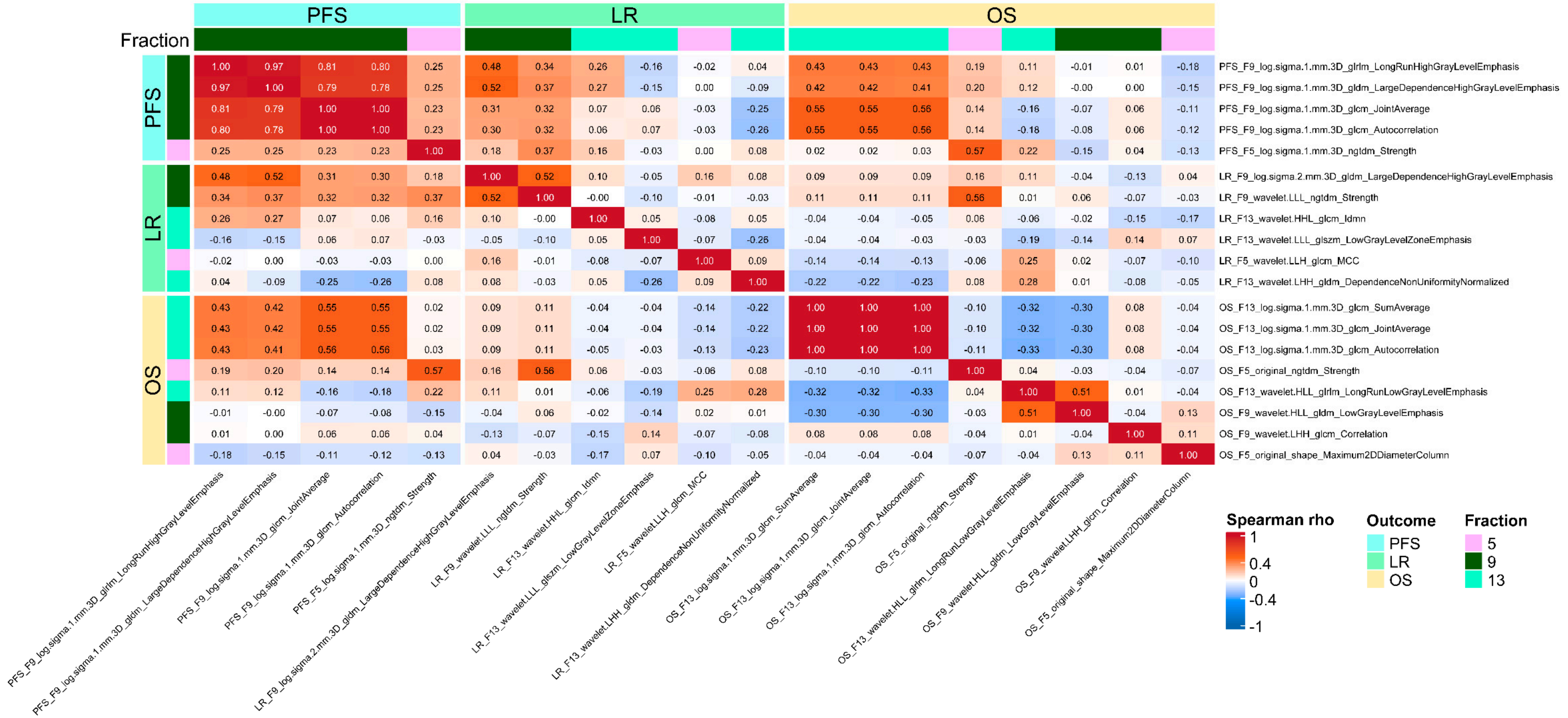


**Supplementary Figure S1.** Correlation heatmap of treatment-fraction-related Δ-radiomic features.
