# Supplementary material for: Longitudinal Delta-Radiomics Identifies Treatment-Fraction-Associated Features Linked to Outcomes After Carbon-Ion Radiotherapy for Head and Neck Cancer
Source: Diagnostics (Basel). 2026 Oct 12;16(20):3309. doi: 10.3390/diagnostics16203309 (PMC13703572; doi:10.3390/diagnostics16203309)
Supplement: Supplementary file 1 [file diagnostics-16-03309-s001.zip › Supplementary Figure S2.pdf]

Outcomes	Binarized Δ-radiomic feature	Subgroup	No. analyzed (events)	Apparent HR (bootstrap interval)	Apparent p-value (bootstrap median)
PFS	log.sigma.1.mm.3D_glrlm_LRHGLE	ACC	43 (5)		0.030 (0.037)
		MM	38 (2)		0.037 (0.039)
		SCC	20 (2)		0.035 (0.036)
PFS	log.sigma.1.mm.3D_gldm_LDHGLE	ACC	43 (5)		0.038 (0.036)
		MM	38 (2)		0.012 (0.013)
		SCC	20 (2)		0.039 (0.035)
PFS	log.sigma.1.mm.3D_glcm_Autocorrelation	ACC	43 (5)	NE	0.126 (0.453)
		MM	38 (2)	NE	0.999 (0.998)
		SCC	20 (2)	NE	0.999 (0.999)
PFS	log.sigma.1.mm.3D_glcm_JointAverage	ACC	43 (5)	NE	0.126 (0.472)
		MM	38 (2)	NE	0.999 (0.998)
		SCC	20 (2)	NE	0.999 (0.999)
OS	wavelet.HLL_glrlm_ LRLGLE	ACC	43 (5)		0.028 (0.037)
		MM	38 (2)	NE	NA
		SCC	20 (2)	NE	NA
OS	original_ngtdm_Strength	ACC	43 (5)	NE	0.173 (0.562)
		MM	38 (2)		0.840 (0.829)
		SCC	20 (2)	NE	0.999 (0.999)
LR	wavelet.LLL_glszm_LGLZE	ACC	25 (5)		0.015 (0.026)
		MM	16 (1)	NE	NA
		SCC	4 (1)	NE	NA
LR	wavelet.LLL_ngtdm_Strength	ACC	38 (5)	NE	0.999 (0.999)
		MM	34 (0)	NE	NA
		SCC	17 (2)		0.639 (0.536)

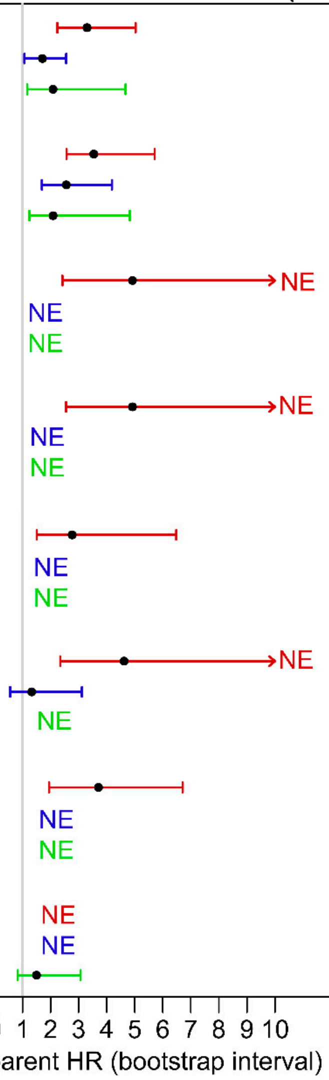

Supplementary Figure S2. Exploratory histology-specific subgroup sensitivity analysis of supported Δ-radiomic features. Subgroup-specific univariable Cox regression was performed within the three histologic subtypes using the cutoff values determined in the full cohort. Points indicate apparent hazard ratios, and horizontal lines indicate bootstrap-derived 2.5th–97.5th percentile intervals. P-values outside parentheses are apparent p-values, and values in parentheses are bootstrap median p-values. NE indicates non-estimable or highly unstable subgroup-specific hazard ratio estimates, including off-scale estimates caused by sparse events. Arrows indicate intervals extending beyond the plotting range. These analyses were exploratory and descriptive because subgroup event numbers were limited, particularly for OS and LR. ACC, adenoid cystic carcinoma; MM, malignant melanoma; SCC, squamous cell carcinoma. LRHGLE, LongRunHighGrayLevelEmphasis; LDHGLE, LargeDependenceHighGrayLevelEmphasis; LRLGLE, LongRunLowGrayLevelEmphasis; LGLZE, LowGrayLevelZoneEmphasis; NE, not estimable.
